# Prevalence and Characteristics of Physiological Gaze-Evoked and Rebound Nystagmus: Implications for Testing Their Pathological Counterparts

**DOI:** 10.3389/fneur.2020.547015

**Published:** 2020-10-22

**Authors:** Michelle Sari Ritter, Giovanni Bertolini, Dominik Straumann, Stefan Yu Bögli

**Affiliations:** ^1^Department of Neurology, University Hospital Zurich, Zurich, Switzerland; ^2^University of Zurich, Zurich, Switzerland; ^3^Clinical Neuroscience Center, Zurich, Switzerland; ^4^Swiss Concussion Center, Schulthess Clinic, Zurich, Switzerland

**Keywords:** video-oculography, nystagmus, cerebellum, gaze-holding, clinical examination, gaze-evoked nystagmus, rebound nystagmus

## Abstract

**Objective:** Cerebellar diseases frequently affect the ocular motor neural velocity-to-position integrator by increasing its leakiness and thereby causing gaze-evoked nystagmus (GEN) and rebound nystagmus (RN). Minor leakiness is physiological and occasionally causes GEN in healthy humans. We aimed to evaluate the characteristics of GEN/RN in healthy subjects for better differentiation between physiological and pathological GEN/RN.

**Methods:** Using video-oculography, eye position was measured in 14 healthy humans at straight ahead eye position before and after, and during 30 s of ocular fixation at 4 horizontal eccentric targets between 30° and 45°. We determined the eye drift velocity and the prevalence of nystagmus before/during/after eccentric fixation.

**Results:** Eye drift velocities during (range: 0.62 ± 0.53°/s to 1.78 ± 0.69°/s) and after eccentric gaze (range: 0.28 ± 0.52°/s to 1.48 ± 1.02°/s) increased with the amount of gaze eccentricity (30°-45°). During continuous eccentric gaze, eye drift velocities decreased by 0.41 ± 0.18°/s at 30°, and 0.84 ± 0.38°/s at 45° gaze eccentricity. GEN was elicited in 71% of subjects at 30° gaze eccentricity. Twenty-one percent showed RN thereafter. This prevalence increased to 100% (GEN)/72% (RN) at 45° gaze eccentricity. RN found after 30° gaze eccentricity was of low velocity (0.82 ± 0.21°/s) and occurred after minor drift velocity decrease during prior eccentric gaze (0.43 ± 0.15°/s).

**Conclusion:** GEN and RN should be tested using horizontal gaze eccentricities of <30°, since most healthy subjects physiologically show GEN and RN at higher eccentricities. In case of an uncertain result, both the reduction of eye drift velocity during eccentric gaze and the velocity of RN can be analyzed to distinguish physiological from pathological nystagmus.

## Introduction

Cerebellar diseases frequently affect the ocular motor neural velocity-to-position integrator. This is a neural network within the brainstem and the cerebellum that generates the position command for the ocular motor neurons to enable stable eccentric gaze (1–4). Already in its normal state, it exhibits some “leakiness” as indicated by centripetal drifts occurring in darkness in healthy individuals attempting eccentric gaze (5, 6). Cerebellar loss-of-function (such as due to drug/alcohol toxicity, malnutrition or cerebellar neurodegeneration/deficits) causes an increase of integrator leakiness thereby leading to gaze-evoked nystagmus (GEN) (1–4, 7–9) and rebound nystagmus (RN) (10–12). GEN is a centrifugal nystagmus occurring at eccentric eye positions, while RN describes a centripetal nystagmus that appears upon the return of gaze to the primary position (straight ahead) after prolonged eccentric gaze.

In clinical neurologic examination, GEN and RN are tested by asking the patient to visually fixate a horizontal eccentric target at roughly 20–30° for up to 20 s (to check for GEN) before asking the patient to look at a subsequent target at primary position (to check for RN). Video-oculography is frequently used for quantification of GEN and RN.

GEN is commonly seen as a clinical sign of a cerebellar lesion, which helps to identify patients with acute vestibular syndrome due to deficits of central vestibular pathways (8, 13). Yet, GEN has frequently been described in healthy subjects with a prevalence of up to 21% already at 10° horizontal gaze eccentricity, increasing up to 93% at extreme lateral gaze (14, 15). RN is also seen as a sign of cerebellar disease. In fact, RN can emerge before any other signs of cerebellar disease are detectable and therefore brain MR-imaging has been recommended in any unexplained case of RN (12, 16). Like GEN, RN has been described in healthy subjects, albeit most commonly only after continuous gaze at larger eccentric gaze angles between 40° and 60° (17–19).

While RN is often examined in daily clinical practice, only few studies have described the characteristics of physiological RN and its relation to physiological GEN. Thus, the aim of this study was to evaluate physiological GEN and RN in healthy human subjects before, during, and after prolonged gaze at different horizontal eccentricities using video-oculography. The results can ease distinguishing physiological GEN or RN from their pathological counterparts.

## Methods

The study protocol was approved by the local ethics committee (cantonal ethics commission Zurich, KEK-ZH-2012-0150) and was in accordance with the ethical standards laid down in the 2013 Declaration of Helsinki for research involving human subjects. Written informed consent was obtained from each subject. Participants with neurologic/psychiatric disease, and subjects who were regularly taking medication were excluded. In total, 14 healthy participants (seven male/seven female, aged 29.7 ± 13.3 years) were included.

### Experimental Setup

All recordings were obtained with the participant seated on a chair with their head being stabilized in upright position using a thermoplastic mask (Sinmed, BV, Reeuwijk, Netherlands). Visual targets were generated by LEDs mounted on a hemispherical screen at 1.5 m distance or a laser projecting to this screen at eye level of the participant. Aside from these visual stimuli, the experiment was conducted in complete darkness. Binocular horizontal eye movements were recorded at 220 Hz with two head-mounted infrared cameras (EyeSeeCam, Munich, Germany). At all times, the left eye was covered using a lens filter, preventing binocular vision but still allowing for recording by the infrared camera. Participants were asked to fixate a flashing red target (20 ms every 2 s, to allow for staying eccentrically without being able to fixate) without moving their head. A total of four trials was conducted per subject. Each trial consisted of three parts. First, “baseline” eye position at the straight ahead horizontal eye position (primary position) was measured for 5 s (step A). Then eye position during 30 s of eccentric gaze was assessed (termed “induction phase,” step B). Immediately afterwards, eye position was measured again at the straight ahead eye position for 10 s (step C). After step C the light in the room was turned on for 60 s to prevent contamination of the following trial from prior testing. In each of the four trials the gaze angle of step B was changed (30°, 35°, 40°, and 45° temporally of the viewing eye), while steps A and C were measured at the straight ahead horizontal eye position. In case of interruption during step B or step C (e.g., missing the target position), the trial was repeated.

### Data Analysis

Data analysis was performed using custom-written interactive programs in MATLAB Version R2016b (The Mathworks, Natick, MA, USA). Velocity traces were obtained as the derivative of horizontal eye position traces. The appearance of GEN and RN was assessed by extracting the occurrence of at least 3 consecutive saccades (within 10 s) in centrifugal (for GEN) or centripetal (for RN) direction with preceding eye drifts in the opposite direction.

Eye drift velocity for steps A and C was calculated as the median eye velocity over a time window of 2–5 s (average of 3.12 ± 1.16 s) after gaze reached the target position (identified by visual inspection). This time window was interactively adjusted depending on data quality and depending on the duration and vigor of the eye drift. This time window was defined for each participant once, and remained the same for all parts of all trials of the same participant. For each trial, the data from both eyes (viewing and non-viewing eye) were pooled as validated by previous works (6, 20). In addition, the median eye velocity at the beginning and the end of eccentric gaze fixation period (step B) was extracted using the same time window as described above (i.e., with a time window of, e.g., 3 s the median of the first 3 s and the one of the last 3 s of the eye drift velocity during step B was calculated). Statistical testing was conducted using SPSS (IBM, Armonk, NY, USA). The data is shown as mean ± standard deviation. One-way repeated measures ANOVA was used to determine the dependence of the eye movements on the eccentricity of the induction phase. The *p*-values were corrected for multiple comparison using Bonferroni adjustment. Principal component analysis was used to evaluate correlations between dependent variables providing the goodness-of-fit (*R*^2^-value), the *p*-value, and the slope of the fit including the 95% confidence interval (CI).

## Results

**Figure 1:**
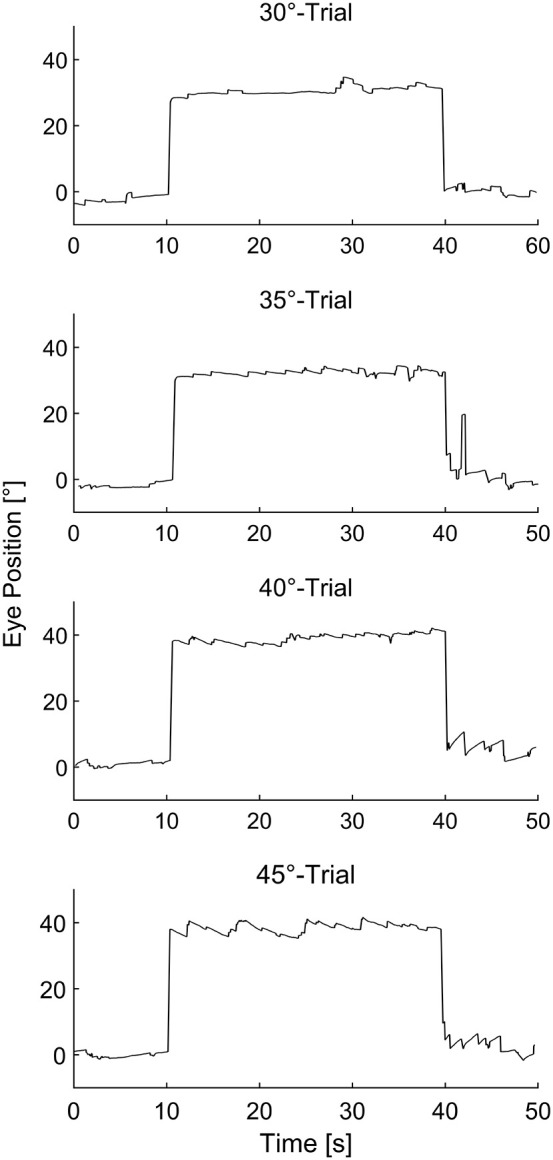
Representative eye movement traces of a single subject before, during, and after sustained eccentric gaze at 30°, 35°, 40°, and 45° respectively.

In this study, we evaluated the appearance of eye drift and physiological nystagmus before, during, and after prolonged horizontal eccentric gaze between 30° and 45° (induction phase) in healthy human subjects. Eye movement traces showing horizontal eye position before, during, and after prolonged eccentric gaze at all tested eccentricities in a representative subject are depicted in Figure 1. Physiological GEN was elicited in all 14 subjects, while physiological RN could only be elicited in 11 subjects. At 30° physiological GEN manifested in 10 participants, while physiological RN only manifested in three participants. These values increased to 10/6 at 35°, 13/11 at 40°, and 14/10 at 45° for physiological GEN and RN, respectively.

**Figure 2:**
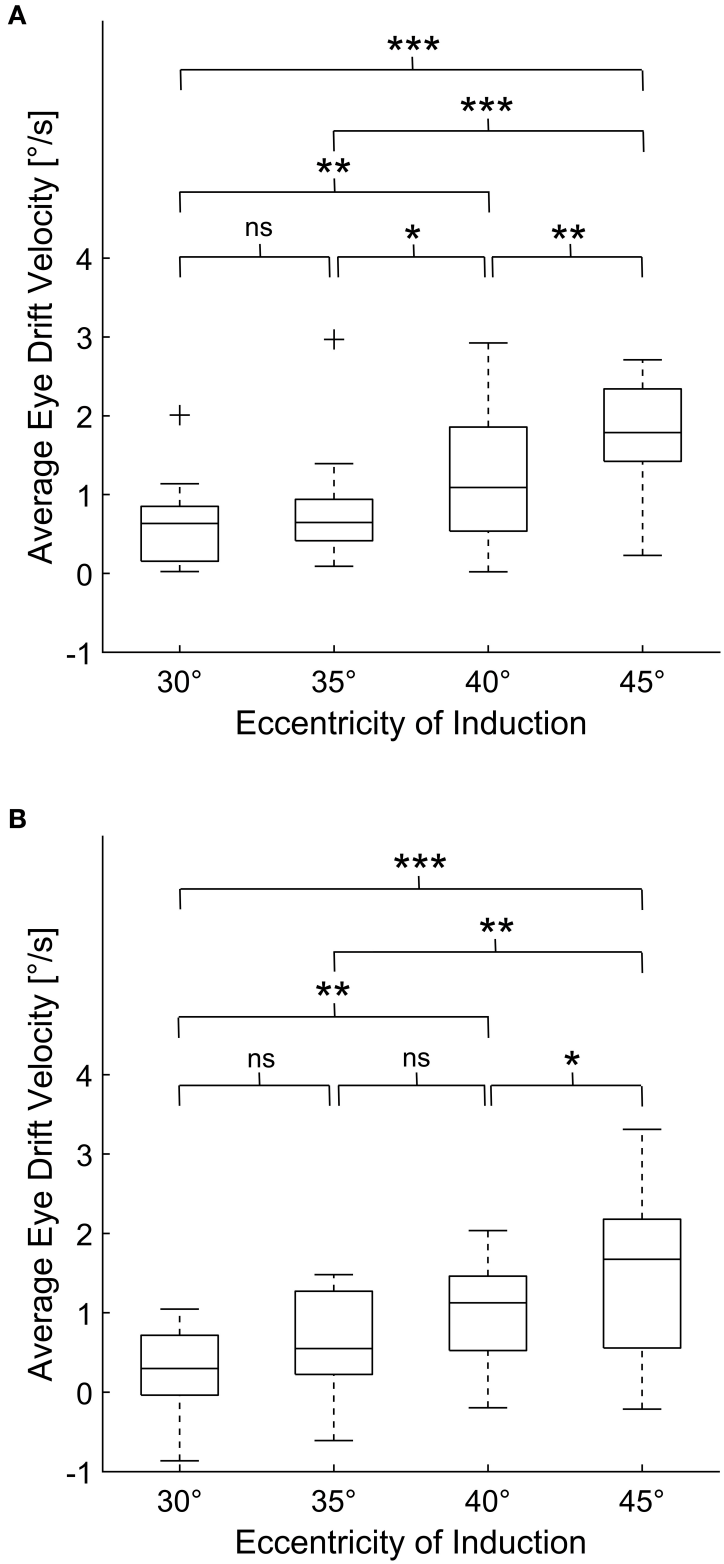
Average eye drift velocities of all subjects during 30 s of eccentric gaze **(A)** and after subsequent return to primary position **(B)**. For each box, the central line indicates the median value. The bottom and top of the box indicate the 25th and 75th percentiles, while the whiskers extend to the most extreme data points that are not considered outliers. The outliers are marked with “+.” One-way repeated measures ANOVA demonstrated statistically significant changes in eye drift velocity depending on the eccentricity of the induction phase for both eye drift during and after 30 s of eccentric gaze. The *p*-values, corrected for multiple comparison using Bonferroni adjustment, are shown in each panel (ns for *p* > 0.05, * for *p* ≤ 0.05, ** for *p* ≤ 0.01, *** for *p* ≤ 0.001).

The mean eye drift velocity at 0° during baseline measurements (i.e., before the induction phase) was 0.03 ± 0.28°/s. The eye drift velocities found at the beginning, during and after the induction phase are summarized in Table 1. Using one-way repeated measure ANOVA statistically significant changes in eye drift velocity could be shown for both the velocity at the beginning of the induction phase [F_(1, 954, 25.407)_ = 26.508, *p* < 0.001] as well as thereafter [F_(3, 39)_ = 16.921, *p* < 0.001]. *Post hoc* analysis revealed the eye drift velocities to increase depending on the eccentricity of the induction phase (Figure 2).

**Table 1:** Absolute eye drift velocity at the beginning, during and after the induction phase.

**Eccentricity of the induction phase**	**Absolute eye drift velocity**		
	**At the beginning of the induction phase**	**During the induction phase (reduction of eye drift velocity)**	**After the induction phase at 0^**°**^**
30°	0.62 ± 0.53°/s	0.41 ± 0.18°/s	0.28 ± 0.52°/s
35°	0.82 ± 0.72°/s	0.58 ± 0.26°/s	0.61 ± 0.61°/s
40°	1.22 ± 0.83°/s	0.70 ± 0.30°/s	0.96 ± 0.65°/s
45°	1.78 ± 0.69°/s	0.84 ± 0.38°/s	1.48 ± 1.02°/s

During prolonged eccentric gaze, the eye drift velocity decreased (Table 1). Yet, physiological GEN persisted during the continuous eccentric gaze holding in 7 (64%), 8 (80%), 13 (100%), 13 (93%) subjects for eccentric gaze at 30°/35°/40°/45°, respectively. Using principal component analysis, a strong relationship between the drop of eye drift velocity during the induction phase and the subsequent emergence of centrifugal eye drift when returning gaze to the primary position was observed in all our subjects [*R*^2^ = 0.82, *p* < 0.001, slope = 0.52 (95%–CI = 0.43–0.67)].

Eye drift did not always result in nystagmus. Therefore, we decided to separately pool subjects who displayed nystagmus and subjects who did not. Subjects who displayed physiological GEN had significantly higher eye drift velocities (1.33 ± 0.75°/s) at the beginning of the induction phase in comparison to those who did not (0.35 ± 0.25°/s, unpaired *t*-test, *p* < 0.001). Furthermore, those subjects who displayed physiological RN showed a significantly higher reduction of eye drift velocity during the induction phase (beginning minus end of induction phase, 0.79 ± 0.33°/s) in comparison to those who did not (0.45 ± 0.20°/s, unpaired *t*-test, *p* < 0.001). In the subjects, in whom physiological RN was elicited already at 30° of eccentric gaze, we measured a RN eye drift velocity of 0.82 ± 0.21°/s which corresponded to the small decrease of eye drift velocity during the induction phase (0.43 ± 0.15°/s).

## Discussion

This study provides a quantitative description of eye drift and physiological RN/GEN before, during, and after prolonged horizontal eccentric gaze at 30–45° in healthy human subjects.

In our study, physiological GEN could be elicited in all tested subjects with the prevalence being dependent on eccentricity (71% at 30° up to 100% at 40–45°). While the prevalence found at extreme eccentric gaze matches prior studies (15, 21, 22), the prevalence at lower eccentricities such as 30–35° was higher [71% in our study vs. 0–58% in prior studies (14, 15, 19)]. Eye drift velocities of physiological GEN were rarely reported. Furthermore, the reported values had large variabilities ranging from as low as 0.26°/s up to 28°/s (6, 21, 22), in comparison to our mean values of 0.62 ± 0.53°/s to 1.78 ± 0.69°/s. Similar to previously reported, velocities of eye drift decreased during continuous eccentric gaze, thus increasing gaze stability (23). Unlike physiological GEN, physiological RN was only found with a prevalence of 21% after eccentric gaze at 30°. The prevalence increased with the eccentricity of prior gaze up to 79% after extreme lateral gaze. This is in contrasts to most prior studies that only described physiological RN after eccentric gaze of 40° or more (17–19, 24), while confirming a single study of five subjects that also found physiological RN after eccentric gaze at 30° (25). Velocities were rarely reported, but if so, ranged between 0.3 and 6.8°/s (24, 25) in comparison to our range of 0.28 ± 0.52°/s to 1.48 ± 1.02°/s.

In comparison to studies performed in patients with cerebellar disease, GEN eye drift velocities found in our study were at the low end of reported velocities (1–24°/s) (9, 11, 23, 26, 27). Eye drift velocities of RN in patients with cerebellar disease were rarely reported. However, the values found in our study were largely lower in comparison to the previously reported range of 2.29–30°/s (23, 28).

Studies on cerebellar disease commonly included patients by mere appearance of nystagmus disregarding the actual drift velocities or a possible correlation between GEN and RN. This is similar to routine clinical examination. Eye drift velocity cannot directly be quantified by eye. Accordingly, the amount of saccades and the repetitive occurrence thereof (which is easily quantifiable) is commonly used for the evaluation of nystagmus. The weakness of this procedure is evident if one considers that GEN already occurred in 71% of subjects at a relatively low eccentricity of 30°. We demonstrate that even persistence of GEN over time is not a solid sign for being pathological. While prior reports described GEN to be pathological if persistent over 20 s (27, 29), we observed that (although the velocity of drift decreased during prolonged eccentric gaze) GEN commonly persisted for the duration of 30 s (in 64–100% of subjects depending on gaze eccentricity). Furthermore, while the velocities found in our study were generally at the low end of ranges presented in prior studies in patients, these results still indicate that certain cases might have been wrongly identified as pathological.

Similar to physiological GEN, physiological RN was also elicited using all induction eccentricities tested. However, this study demonstrates differences that can be used to distinguish physiological from pathological RN. In those cases where physiological RN was elicited after eccentric gaze at 30°, velocities were largely lower than in patients [average of 0.82 ± 0.21°/s in our study vs. previously reported range of 2.29–30°/s (23, 28)]. Secondly, the amount of adaptation that occurred prior to the appearance of physiological RN during eccentric gaze may be an important sign to be observed, as it was much lower than the values reported in a previous study using a similar methodology in patients with cerebellar disease (average of 0.43 ± 0.15°/s in our study vs. 2.40°/s) (23).

The exact mechanism of RN and its relation to GEN are unknown. In this study, we found a high correlation between the decrease of physiological GEN and subsequent physiological RN. This finding supports current hypothesis suggesting that during sustained eccentric gaze, different adaptive mechanisms alter the neural integrator to enhance gaze stability at this eccentric gaze position by decreasing the centripetal eye drift by a change of the set-point (gaze angle with least eye drift), which then causes a gaze-instability at primary position (23–25), altogether suggesting that RN and GEN are physiological phenomena of the same neural integrator that are exacerbated by cerebellar disease.

## Limitations

Our study has several limitations. Firstly, we only tested 14 subjects, which is a rather small sample size. Secondly, parameters used during our study are different from parameters that are generally used during bedside examination. These include the duration of eccentric gaze (this study: 30 s, bedside: maximally 20 s), the horizontal eccentricity of gaze (this study: 30–45°, bedside: 20–30°), the prevention of fixation (this study: experiment conducted in darkness with one eye covered, bedside: binocular vision in a lit room) and the fixation target (this study: flashing target appearing for 20 ms every 2 s, bedside: continuously visible target). Thus, no direct correlation to the clinical examination is possible. However, the parameters used in this study are known to provoke occurrence of nystagmus. The occurrence of only minor physiological RN after eccentric gaze at 30° for 30 s is thus of high importance. The results improve the significance of any RN that is found during general clinical examination with parameters that are easier (e.g., less gaze eccentricity, reduced duration of eccentric gaze, improved fixation) than in this study. Lastly, methods used during the testing were similar, however not the same as in prior reports, thus comparisons have to be made cautiously. Direct comparisons can only be made with one study (23) that used the same methods testing patients with cerebellar degeneration. In the future further studies directly comparing patients and matched healthy subjects using the same paradigms are necessary.

## Conclusions

Our study provides an in-depth description of eye drift and physiological nystagmus before, during, and after prolonged eccentric gaze at eccentricities between 30° and 45° in healthy. We provide values for physiological eye drift velocities that can and should be expected during an examination of eye movements/nystagmus using video-oculography. Furthermore, our results show that GEN and RN should be tested at eccentricities of <30° using video-oculography, as higher eccentricities lead to significant gaze drift and physiological nystagmus (most commonly GEN) in healthy subjects, while pathological nystagmus can often already be elicited at lower eccentricities (4, 9, 11, 23). In case of an uncertain result both the reduction of GEN drift velocity (adaptation) during eccentric gaze and the drift velocity of RN can be analyzed to distinguish physiological from pathological nystagmus, while the occurrence and persistence of GEN during continuous eccentric gaze are not *per se* pathological.

## Data Availability Statement

The raw data supporting the conclusions of this article will be made available by the authors, without undue reservation.

## Ethics Statement

The studies involving human participants were reviewed and approved by Kantonale Ethikkommission Zürich Stampfenbachstrasse 121, 8090 Zürich, Switzerland. The patients/participants provided their written informed consent to participate in this study.

## Author Contributions

GB, DS, and SB conceived and designed the study. MR and SB performed the experiments and acquired the data. MR, GB, and SB analyzed the data. MR and SB drafted the manuscript. All authors interpreted the data and revised the manuscript for intellectual content and approved the submitted version.

## Conflict of Interest

The authors declare that the research was conducted in the absence of any commercial or financial relationships that could be construed as a potential conflict of interest.

## Abbreviations

GEN: gaze-evoked nystagmus
RN: rebound nystagmus.
